# Coexistence of Hashimoto’s Thyroiditis in Differentiated Thyroid Cancer: Post-Operative Monitoring of Anti-Thyroglobulin Antibodies and Assessment of Treatment Response

**DOI:** 10.3390/diagnostics14020166

**Published:** 2024-01-11

**Authors:** Alessandra Donnici, Maria Mirabelli, Stefania Giuliano, Roberta Misiti, Vera Tocci, Marta Greco, Vincenzo Aiello, Francesco S. Brunetti, Eusebio Chiefari, Antonio Aversa, Daniela P. Foti, Antonio Brunetti

**Affiliations:** 1 Endocrinology Unit, University Hospital “Renato Dulbecco” of Catanzaro, 88100 Catanzaro, Italyaversa@unicz.it (A.A.); 2 Department of Health Sciences, University “Magna Græcia” of Catanzaro, 88100 Catanzaro, Italy; 3 Clinical Pathology Unit, University Hospital “Renato Dulbecco” of Catanzaro, 88100 Catanzaro, Italymarta.greco@unicz.it (M.G.); 4 Department of Experimental and Clinical Medicine, University “Magna Græcia” of Catanzaro, 88100 Catanzaro, Italy; 5 Rheumatology Clinic ‘Madonna dello Scoglio’ Cotronei, 88900 Crotone, Italy

**Keywords:** Hashimoto’s thyroiditis, thyroid autoimmunity, differentiated thyroid cancer, anti-thyroglobulin antibodies

## Abstract

Introduction: Differentiated thyroid carcinoma (DTC) is frequently found in conjunction with autoimmune thyroid disorders, particularly Hashimoto’s thyroiditis (HT). This study investigates the impact of coexisting HT on the persistence of an indeterminate response to therapy due to positive anti-thyroglobulin antibodies (AbTg), measured via competitive immunoassay, in a consecutive patient series from Calabria, Southern Italy. Methods: This retrospective longitudinal study analyzed 259 consecutive DTC patients managed at the Endocrinology Unit of Renato Dulbecco Hospital (Catanzaro, Italy) up to 2023. Patients with medullary and undifferentiated thyroid carcinoma, partial thyroidectomy, less than six months of post-operative monitoring, or missing clinical data were excluded. Demographic information, histological findings, initial tumor stage, and ATA risk category were collected. The response to therapy was assessed based on ATA guidelines. Results: Among the 259 patients, 29% had coexisting HT. Patients with HT exhibited distinct characteristics: a higher proportion of females (87.0% vs. 74.7%), a shorter post-operative monitoring duration (median 3 vs. 5 years), and a higher prevalence of papillary thyroid carcinoma (PTC) (97.4% vs. 86.3%). The tumor size, lymph node involvement, and distant metastasis were similar between the groups, with patients without HT having a higher incidence of extrathyroidal tumor extension. However, the initial TNM stage and ATA risk category did not differ significantly. At the six-month follow-up, HT patients showed a higher rate of indeterminate responses, primarily due to positive AbTg. After 12 months, the response categories aligned, with decreasing AbTg levels in the HT group. After 24 months, most patients with long-term follow-up demonstrated an excellent response to DTC therapy, irrespective of HT coexistence. Conclusions: While HT does not worsen DTC prognosis, it may result in indeterminate responses. AbTg measurements in the peri-operative period should be encouraged to facilitate post-operative monitoring, emphasizing the importance of using standardized assays. Further research in larger populations with extended follow-up is needed to comprehensively understand the HT-DTC relationship.

## 1. Introduction

It is estimated that around 25% of patients diagnosed with differentiated thyroid carcinoma (DTC) also exhibit coexisting autoimmune thyroid disorders, primarily Hashimoto’s thyroiditis (HT) [1]. The potential correlation between HT and malignant thyroid neoplasms, especially papillary thyroid carcinoma (PTC), was initially reported in 1955. Since then, a growing body of evidence has substantiated this association [2,3]. Notably, a recent meta-analysis demonstrated a statistically significant relative risk of HT in 11,155 PTC patients (relative risk: 2.36) and a relative risk of PTC in 7873 HT patients (relative risk: 1.4) [4]. HT is an organ-specific autoimmune condition involving immune-mediated thyroid destruction with B and T lymphocyte infiltration. The resulting autoimmune response entails cell-mediated processes, leading to the production of anti-thyroid autoantibodies as markers of follicular damage rather than direct cytotoxic agents [5,6,7]. Three proposed pathogenetic mechanisms could explain the coexistence of HT with DTC: (i) chronic inflammation, fostering malignant cell transformation through pro-inflammatory cytokines and growth factors; (ii) elevated TSH levels in HT patients progressing to hypothyroidism may stimulate thyroid epithelial cell proliferation; and (iii) a common genetic background, including RET/PTC gene rearrangement and p53 mutations, may also contribute to this association [8,9]. Despite HT being suggested as a potential risk factor for the development of DTC, there is evidence indicating that individuals with HT tend to exhibit less aggressive clinical and pathological tumor characteristics at the time of diagnosis and experience better prognoses [10]. Potential explanations for this phenomenon include increased thyroid ultrasound assessments among HT patients, facilitating early DTC identification, or the lymphoplasmacytic infiltrate potentially constraining tumor cell proliferation [11]. In recent years, there has been an increase in the incidence of thyroid cancer. This is mainly due to the detection of small and less severe tumors (microcarcinomas), as well as the discovery of incidental DTC during surgeries performed for non-cancer-related reasons [12,13]. The COVID-19 pandemic may also have contributed to this surge, as Italy has seen a rise in new thyroid cancer cases among patients who have overcome the viral infection [14,15]. Managing DTC with coexisting HT presents clinical and laboratory challenges, especially in interpreting thyroglobulin (Tg) levels in the presence of positive anti-thyroglobulin antibodies (AbTg). The presence of positive AbTg antibodies has a significant impact on the accuracy of Tg measurements, resulting in either an overestimation or underestimation of Tg levels depending on the assay method used [16,17]. Additionally, the threshold level at which interference occurs is still being debated [18]. As a result, the guidelines of the American Thyroid Association (ATA) recommend measuring AbTg antibodies in post-operative assessments in DTC. Even with very low Tg levels, the presence of positive AbTg antibodies can affect the interpretation of the treatment response, which is defined as indeterminate instead of excellent [19]. In this regard, a recent multicentric observational study conducted in Italy has found an increased risk of non-excellent responses to thyroidectomy (+/− radioactivùe iodine therapy, RAI) in patients with HT compared to those without HT. However, the study only followed DTC patients for one year and did not include data from the Calabrian population [20]. Currently, no studies have explored the relationship between HT and DTC treatment response in Calabria. It is worth noting, however, that over the last two decades, this southern Italian region has undergone notable shifts in the landscape of thyroid disorders due to changes in iodine nutritional status and a substantial increase in autoimmune thyroid conditions [12,13,21]. Therefore, the purpose of this study was to investigate the influence of coexisting HT on long-term treatment responses in DTC in a Calabrian patient cohort followed-up at our institution. Moreover, the assessment of their post-operative AbTg levels could potentially contribute to refining the current clinical recommendations for the laboratory monitoring of DTC when HT coexists.

## 2. Materials and Methods

### 2.1. Study Population

**Figure 1:**
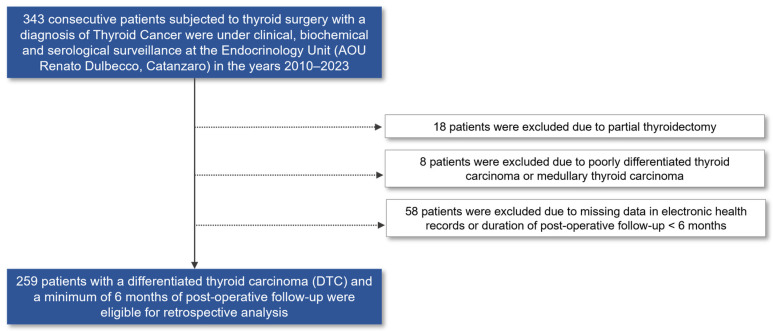
Flowchart of the study.

For this retrospective longitudinal study, all the consecutive surgical patients with thyroid cancer (including incidental micro- and macro-DTC) who had been managed at the tertiary care Renato Dulbecco Hospital of Catanzaro, Italy (Endocrinology Unit) from 2010–2023 were assessed for participation eligibility. Exclusion criteria were histological diagnosis of medullary thyroid carcinoma (MTC); undifferentiated thyroid carcinoma (UTC), partial thyroidectomy as the surgical strategy of choice for the management of DTC; a duration of follow-up shorter than 6 months; and missing clinical data in the electronic health records. Figure 1 shows the participant selection workflow that was followed for our retrospective analysis. Medical records of eligible participants with DTC, including PTC, follicular thyroid carcinoma (FTC), and Hürthle cell carcinoma (HCC) histology, according to the WHO classification (4th edition) [22], who underwent total thyroidectomy and at least 6 months of follow-up visits, were reviewed by trained endocrinologists. Retrospectively collected data included patient demographics, histological findings at the time of diagnosis (i.e., maximum tumor size, tumor focality, DTC histology, extrathyroidal extension, cervical lymph node status when dissection was performed), initial tumor stage (according to AJCC/TNM 8th edition [23]), and ATA risk category (low, intermediate, or high) according to malignant tumor features and information on post-operative adjuvant RAI administration. The administration of post-operative RAI therapy followed the ATA guidelines [19]. Patients with low-risk T1a (as per the AJCC/TNM staging system) DTC did not systematically receive RAI therapy, while those with higher tumor stages were recommended to undergo adjuvant RAI, in consideration of histological characteristics and patient-related factors. The doses of RAI administered did not generally exceed 100 mCi per cycle. Furthermore, duration of follow-up and detailed information on response to therapy, based on periodical neck ultrasound and laboratory evaluations and/or additional imaging findings, were collected in order to assess the study outcomes. Eligible patients for inclusion were divided into two distinct groups based on a pre-operative clinical diagnosis of HT, as defined by suggestive US findings and/or positivity to anti-thyroid markers, with or without hypothyroidism; unspecified hypothyroidism in the presence of a family history of HT; or providing a valid external hospital certificate proving HT-related ticket exemption. In cases of thyroid nodular disease, relevant information regarding the indication for thyroidectomy was also collected, which was performed when there was suspicion of malignancy based on the pre-operative fine needle aspiration (FNA) results of TIR 3b, TIR 4, or TIR 5 [24]. This patient cohort has been involved in another observational investigation addressing the long-term risk of persistence or relapse of DTC [25], but the data were independently analyzed.

### 2.2. Outcome Assessment and Laboratory Determinations

Response to DTC therapy was assessed retrospectively in a longitudinal manner, using the categories of excellent, indeterminate, biochemically incomplete, and structurally incomplete, as defined by ATA guidelines. This assessment was conducted at 6, 12, and 24 months after surgical treatment with or without post-operative adjuvant RAI administration, as well as at the last available follow-up visit, using data from medical records. Clinical and ultrasound evaluations were performed as previously described [25]. Laboratory determinations of basal or stimulated serum Tg were performed at the Clinical Pathology Unit. The assays used for analysis were LIAISON^®^ Tg II Gen assay (DiaSorin, S.p.A., Saluggia, Italy), a 2-step sandwich chemiluminescence immunoassay using magnetic particles coated with a mixture of mouse monoclonal antibodies and monoclonal antibodies linked to an isoluminol derivative (CV% intra-assay ranging from 2% to 4.4% and inter-assay variation ranging from 3.5% to 4.4%) and, for the dosage of AbTg, the ADVIA Centaur^®^ (Siemens Healthcare Diagnostics Inc., Camberley, UK) Anti-thyroglobulin (aTg), a competitive immunoassay, which uses direct chemiluminescent technology (intra-assay CV% ranging from 3.4% to 5.8% and inter-assay CV% ranging from 6.6% to 6.2%). The assays have been calibrated using, respectively, the Reference Standard CRM 457 [26,27] and the WHO (World Health Organization) Reference Standard MRC 65/93 [28] for Tg and for AbTg measurements. An excellent response indicated the absence of any clinical, biochemical, or ultrasound evidence of the disease, with basal serum Tg levels below 0.2 ng/mL or stimulated serum Tg levels below 1 ng/mL, and negative AbTg antibodies. An indeterminate response indicated a situation where residual or recurrent disease could not definitively be excluded due to a mild elevation of serum Tg (0.2–1 ng/mL for basal Tg or 1–10 ng/mL for stimulated Tg), stable or declining positive AbTg antibodies, and/or nonspecific imaging findings. A biochemically incomplete response referred to a significant elevation of serum Tg (above 1 ng/mL for basal Tg or above 10 ng/mL for stimulated Tg) without structural evidence of disease on neck ultrasound or other imaging tests. A structurally incomplete response indicated the presence of disease on neck ultrasound or other imaging tests, typically accompanied by a significant elevation in basal serum Tg or positive AbTg antibodies. In this study, patients who had an indeterminate response to DTC therapy at two consecutive follow-up visits were classified as having a “persistence of indeterminate response”, regardless of their pre-operative HT status. Patients who transitioned from an excellent or incomplete response to an indeterminate response at a specific follow-up visit were classified as having a “new onset indeterminate response”. Only one patient in the selected study population died as a result of DTC.

### 2.3. Statistical Analysis

The data were presented as either the median and interquartile range or as count and percentage. Differences between patients with a positive and negative pre-operative HT status were assessed using the Mann–Whitney test for continuous variables and the chi-squared test for categorical variables. Fisher’s exact test was used to compare the frequencies of uncommon outcomes, such as the persistence of an indeterminate response to treatment at 12 and 24 months of follow-up. Logistic regression was employed to identify predictive factors for the persistence of an indeterminate response to DTC therapy, with the strength of association estimated as adjusted odds ratios and 95% confidence intervals. A significance level of less than 0.05 was used for all statistical tests. The analyses were conducted using the statistical software JASP ver. 0.17.2 (University of Amsterdam, Amsterdam, The Netherlands), based on the R programming language (https://jasp-stats.org/ [Last accessed: 1 January 2024]).

## 3. Results

### 3.1. Characteristics of Patients with Differentiated Thyroid Cancer (DTC) Compared by Pre-Operative Hashimoto’s Thyroiditis (HT) Status

As shown in Figure 1, a total of 259 patients who underwent total thyroidectomy for DTC were eligible for inclusion in this study. All participants were clinically and serologically monitored for a minimum of 6 months following surgical treatment. The clinical characteristics of these patients are presented in Table 1. Among them, 77 patients (29%) had been diagnosed with HT prior to surgery. Female patients were more prevalent in the HT-positive group that in the HT-negative group (87.0% vs. 74.7%, *p* = 0.028). Additionally, patients with HT had a shorter duration of post-operative follow-up, possibly due to the increasing incidence of HT and incidental micro-DTC observed in recent years in Calabria and other Italian regions. There was no significant difference in the age at DTC diagnosis between the two groups (median age: 43 vs. 46 years, *p* = 0.058), although there was a tendency for patients with HT to be diagnosed at a younger age. Tumor histology also differed between the two groups, with a higher prevalence of PTC in patients with HT (97.4% vs. 86.3%, *p* = 0.026) compared to those without HT. The two groups had similar tumor size at diagnosis of DTC (median 11.0 vs. 13.5 mm, *p* = 0.408), as well as tumor focality and prevalence of lymph node and distant site metastasis. The occurrence of distant site metastasis at the time of DTC diagnosis was infrequent, with only three patients demonstrating lung and/or skeletal metastasis (in two cases, along with cervical lymph node involvement). These metastases were exclusively observed in the HT-negative group of patients. Furthermore, patients without HT had a significantly higher occurrence of extrathyroidal extension of DTC. Nonetheless, the majority of patients in both groups were classified as stage 1 according to the AJCC/TNM 8th edition (96.1% vs. 95.1%, *p* = 0.741) and at low risk according to the ATA risk categorization system (64.9% vs. 56.0%, *p* = 0.409), indicating a favorable prognosis. Adjuvant RAI treatment was required in a similar proportion of patients to effectively control the disease, although patients without HT tended to receive more than one cycle of RAI, with cumulative doses typically not exceeding 600 mCi, and four cases required target therapy with tyrosine kinase inhibitors (TKIs). None of the patients with HT required TKIs for oncological management.

### 3.2. Outcomes of DTC by Pre-Operative HT Status and Predictors of Persistence of Indeterminate Response

**Figure 2:**
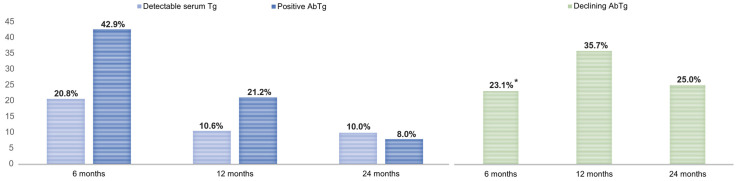
Changes over time in the percentage of HT-positive patients with detectable serum thyroglobulin (Tg) levels or positive anti-thyroglobulin antibodies (AbTg). Light-blue histograms indicate the percentages of HT-positive patients with a mild elevation in serum Tg (i.e., >0.2 ng/mL for basal Tg; >1 ng/mL for stimulated Tg) at each time point in post-operative monitoring. Dark-blue histograms indicate the percentages of patients with positive AbTg levels at each time point in post-operative monitoring. Green histograms indicate the relative percentages of patients who have tested positive for AbTg and have experienced a decline of more than 50% in their AbTg levels from the previous measurement. This reduction has led to a reclassification of their treatment response from indeterminate to excellent. “*” refers to the proportion of patients with positive AbTg levels measured shortly before the thyroidectomy and histological diagnosis of DTC. For this subgroup of patients (*n* = 13), the pre-operative period is used as the baseline for evaluating their decline in AbTg levels during the first six months of post-operative monitoring.

Subsequently, we conducted a longitudinal analysis to compare the outcomes of DTC treatment between the two groups. The results, as displayed in Table 2, indicate a significant difference in the response to DTC treatment at the 6-month post-operative follow-up between patients with and without HT. Specifically, almost half of the DTC patients with HT exhibited an indeterminate response to treatment (45.4% vs. 18.7%, *p* < 0.001), primarily due to the presence of positive AbTg at the 6-month follow-up (43.4%). Only a small portion of the indeterminate response could be attributed to a slight elevation in serum thyroglobulin levels. However, in the case of most patients with HT, there were no available pre-operative measurements of AbTg levels to evaluate their trend. This is because AbTg testing is not a routine procedure for managing thyroid nodules prior to surgery or for monitoring the progression of HT after diagnosis. Furthermore, AbTg do not predict thyroid dysfunction (i.e., hypothyroidism), and it remains uncertain whether these antibodies contribute to the development of PTC within the context of autoimmune thyroiditis or whether they are linked with tumor prognosis [29]. However, when we specifically examined the small subset of patients who had available pre-operative AbTg determinations, measured within 3 months from the histological diagnosis of DTC (*n* = 13), we noted that approximately 25% of them experienced a considerable decline in their AbTg levels as early as 6 months after surgery (Figure 2). Additionally, at the 12-month follow-up, both groups saw a significant reduction in the number of patients with an indeterminate response to DTC therapy, along with a corresponding increase in the number of patients with an excellent response. The reduction in indeterminate cases was particularly notable in the group with a pre-operative positive HT status, decreasing from 45.4% at 6 months to 13.6% at 12 months. Furthermore, after 24 months of follow-up, during which most recurrences are expected in low-risk thyroid tumors [30], there were no significant differences between the two groups in terms of the proportion of different ATA types of response to DTC treatment. Additionally, most patients with HT who had very long-term follow-up showed an excellent response to DTC therapy at the last post-operative visit (Table 2). Overall, these findings suggest that patients with HT experience a decrease in AbTg levels shortly after undergoing surgery, and the outcomes of DTC are comparable or potentially more favorable in the presence of autoimmune thyroiditis.

Finally, in our investigation of factors that may predict a persistent indeterminate response to DTC therapy after 12 months of follow-up, neither a pre-operative positive HT status, tumor histology and size, nor patient age or adjuvant RAI showed significant associations with this type of response. The only predictor found to have a significant positive association was male sex, with an odds ratio of 13.355, as presented in Table 3. However, it is important to note that the estimate’s confidence interval was wide due to the limited sample size of the study.

## 4. Discussion

The debated relationship between HT and DTC has been explored for decades, with studies suggesting a potential association, particularly with PTC [31]. While the exact nature of this relationship is still under investigation, there is evidence to suggest that HT may lead to less severe initial DTC diagnoses and a more favorable prognosis for tumor persistence or progression after one year [32]. Among the consecutively enrolled patients from Calabria included in our study who were diagnosed with DTC, 29% had a coexisting HT as well, thereby supporting the notion of a potential link between HT and the occurrence of thyroid malignancies, as observed in other populations with different genetic background and, possibly, iodine nutrition status [4].

The previous literature has reported a higher proportion of females in cases of DTC coexisting with HT [32], which is also consistent with our findings (87% of female patients in the DTC HT-positive group compared to 74.7% in the DTC HT-negative group). This female predominance may be explained by the impact of circulating estrogens, as previous studies have shown that they enhance the expression of estrogen receptor alpha in cells in papillary thyroid carcinoma, leading to an increased cell proliferation [33]. While HT patients showed a higher prevalence of PTC, in our study, the pathological characteristics of thyroid cancer were comparable between the HT-positive and HT-negative groups. Both exhibited similar tumor sizes and rates of lymph node involvement, with distant metastasis being rare occurrences. However, the group without HT had a higher incidence of minimal extra-thyroidal tumor extension, which is a topic of ongoing debate regarding its influence on cancer outcomes [34]. Despite this difference, there were no significant variations between the two groups in terms of the initial TNM disease stage and ATA risk category.

According to our results, the occurrence of HT and DTC together does not increase the likelihood of persistence/recurrence of cancer. When considering the long-term outcomes of DTC, our analysis suggests that HT either has no effect or may even have a protective influence, which is consistent with findings in other populations [2,3,4,5,6,7,8,9]. This includes the recent 12-month multicentric report of the Italian Thyroid Cancer Observatory, documenting a comparable risk of structural persistent disease among DTC patients with and without HT, notwithstanding an increased occurrence of biochemical incomplete responses in the HT-positive group [20]. Here, for the first time, the differences in treatment responses in DTC were examined at various time intervals (6, 12, 24, and >24 months) in a patient cohort specifically from Calabria. The results from the 6-month visit after surgery revealed a notable difference between the two groups in terms of the percentages of indeterminate responses. Specifically, 45% of patients in the HT-positive group showed an indeterminate response, compared to 18.7% in the HT-negative group. This difference can be attributed to the higher likelihood of patients with HT to have positive AbTg following thyroid gland removal, as AbTg levels tend to decline slowly after surgery (the estimated half-life of AbTg is 10 weeks [35]). However, after 12 months of total thyroidectomy, with or without additional RAI treatment, there was a significant (>50%) reduction in the prevalence of patients with positive AbTg antibodies, resulting in similar rates of indeterminate response between the HT-positive and HT-negative groups. In the HT-positive group, the decline in AbTg levels continued after 24 months post surgery, with only four HT-positive patients still exhibiting detectable AbTg levels. Among these cases, there was a significant trend of reduction compared to a previous assessment. Ultimately, at the end of the follow-up period, none of the patients with HT showed a biochemically incomplete response, and only one patient had a persistent indeterminate response, due to a slight elevation in basal serum Tg and detectable AbTg antibodies.

**Figure 3:**
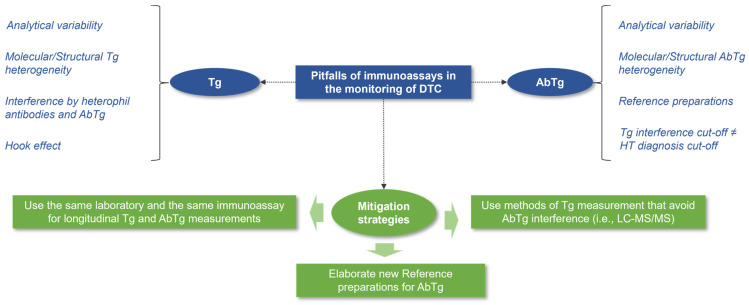
Pitfalls of immunoassays in the post-operative monitoring of DTC. The figure depicts the prevailing constraints when employing immunoassays to measure Tg and AbTg levels, along with potential strategies to mitigate these limitations.

The accurate interpretation of treatment response in DTC during follow-up relies on standardized and consistent measurements, not only of serum Tg but also of AbTg [36]. Immunoassays, now used in most clinical laboratories for Tg determination, while analytically sensitive and specific, face challenges such as a lack of standardization, immunoreactivity, variable sensitivity, heterophil antibody interference, and the hook effect (Figure 3) [37,38,39,40,41]. However, the main limitation is susceptibility to interferences from circulating antibodies, leading to falsely low/undetectable Tg or falsely high Tg [42]. The laboratory monitoring of DTC is further complicated by inter-assay differences, the molecular/structural heterogeneity of Tg (i.e., due to glycosylation and/or iodination), and the in vivo interference of AbTg on the clearance dynamics of Tg [42,43,44]. Commercially available assays for AbTg detection also show varying results, emphasizing the need for new standardized reference preparations [44,45,46,47,48]. The reference preparations for AbTg are outdated [26,27], and manufacturers’ recommended cut-offs for the diagnosis of HT and other autoimmune thyroid disorders may be too high to exclude AbTg interference in Tg immunoassays. Therefore, using the assays’ limit of quantification (LoQ) has been proposed for AbTg interference evaluation [49,50]. Given these pitfalls of immunoassays, performing both Tg and AbTg determinations in the same laboratory should be preferred for the longitudinal monitoring of DTC. Moreover, due to the increasing incidence of DTC in patients with HT, it is advisable to conduct AbTg determination immediately before, or shortly after, the thyroidectomy (i.e., within 10 weeks) to establish a baseline measurement and identify any significant decline in AbTg levels over time. This approach may help reduce the number of indeterminate responses at the 6-month visit and potentially reduce the inappropriate use of RAI, which can have adverse effects on patients without necessarily impacting their cancer-specific survival in the absence of high-risk DTC [51,52]. It is important to inform patients with HT who show indeterminate responses that most disease recurrences in low- to intermediate-risk DTC typically occur in the neck region and can potentially be detected through ultrasound [53]. Therefore, administering RAI solely for easier monitoring of thyroglobulin levels should not be recommended. In relation to this matter, it is important to consider that levels of AbTg may increase or return to positive values within six months after receiving RAI treatment. This phenomenon is predominantly caused by the release of antigens from any remaining thyroid tissue and should not be automatically interpreted as a recurrence of cancer [54,55,56].

The study is subject to several limitations, including its retrospective design, a relatively small sample size, and the absence of strict, histologically based, diagnostic criteria for HT. However, the work provides valuable real-world insights into the management of DTC. Furthermore, the single-center study design mitigates potential discrepancies in Tg and AbTg measurements arising from different laboratories, thereby enhancing the validity of these findings. Notably, the extended observation period of the study yields long-term treatment response data for DTC, both with and without HT coexistence.

## 5. Conclusions

The coexistence of HT does not impact the prognosis of DTC. However, in patients with DTC and HT, there is a higher risk of encountering an indeterminate response, even in the absence of residual or recurrent disease, during the initial six months of post-operative monitoring. This is due to the potential interference of detectable AbTg antibodies. For certain patients (i.e., those being considered for surgery for suspected DTC at pre-operative FNA, especially females or individuals with a positive history or predisposition to autoimmune thyroid disease), the early measurement of AbTg levels could improve post-operative monitoring. It is important to have standardized and consistent methods of measuring AbTg in order to easily identify significant increases or decreases in AbTg levels that may impact the correct classification of treatment responses for DTC.

## Figures and Tables

**Table 1:** Characteristics of the study population affected by differentiated thyroid carcinoma (DTC) divided into two distinct patient groups according to their pre-operative Hashimoto’s thyroiditis (HT) status.

Characteristics	HT-Negative (n = 182)	HT-Positive (n = 77)	*p*
Females, *n*	137 (74.7%)	67 (87.0%)	**0.028**
Age at diagnosis, years	46 (36–57)	43 (32–51)	0.058
Follow-up duration, years	5 (2–10)	3 (1–6)	**0.001**
Thyroidectomy was performed due to a cytologically suspicious nodular disease †, *n*	76 (58.9% *)	30 (46.1% *)	0.092
TIR 3b	26 (34.2%)	8 (26.7%)	
TIR 4	31 (40.8%)	14 (46.6%)	
TIR 5	19 (25.0%)	8 (26.7%)	
DTC histology, *n*			**0.026**
PTC	157 (86.3%)	75 (97.4%)	
High risk PTC subtypes **	9 (5.7% of all PTCs)	9 (11.7% of all PTCs)	
FTC	17 (9.3%)	1 (1.3%)	
HCC	8 (4.3%)	1 (1.3%)	
Tumor size, mm	13.5 (8.0–19.5)	11.0 (8.0–18.0)	0.408
Tumor size category, *n*			
T1a	61 (33.5%)	32 (41.5%)	
T1b	53 (29.1%)	24 (31.2%)	
T2	15 (8.3%)	10 (13.0%)	
T3a	4 (2.2%)	2 (2.6%)	
T3b	49 (26.9%)	9 (11.7%)	
T4a	0 (0%)	0 (0%)	
T4b	0 (0%)	0 (0%)	
ETE, *n*	72 (39.6%)	17 (22.1%)	**0.007**
Minimal ETE	23 (31.9% of all ETE)	8 (47.1% of all ETE)	
Multifocality, *n*	38 (30.9%)	18 (23.4%)	0.655
Lymph node metastasis, *n*	28 (15.4%)	11 (14.3%)	0.821
Distant site metastasis, *n*	3 (1.6%)	0 (0%)	0.257
AJCC/TNM stage, *n*			0.741
Stage 1	173 (95.1%)	74 (96.1%)	
Stage 2	3 (1.6%)	2 (2.6%)	
Stage 3	2 (1.1%)	1 (1.3%)	
Stage 4a	2 (1.1%)	0 (0%)	
Stage 4b	2 (1.1%)	0 (0%)	
ATA risk stage, *n*			0.409
Low	102 (56.0%)	50 (64.9%)	
Intermediate	52 (28.6%)	18 (23.4%)	
High	28 (15.4%)	9 (11.7%)	
Adjuvant RAI, *n*	123 (67.6%)	44 (57.1%)	0.202
>1 cycle	16 (13.0% of all RAI)	2 (4.5% of all RAI)	
Adjuvant TKI therapy, *n*	4 (2.2%)	0 (0%)	0.321
Pre-operative positive AbTg antibodies, *n*	_	13 (16.9%)	

Data are presented as numbers (*n*) and percentages (%), or as medians and interquartile ranges. Comparisons between groups were performed using the chi-squared/Fisher exact test or the Mann–Whitney test, as appropriate. Bold values denote statistical significance at the *p* < 0.05 level. “†” indicates thyroid nodules with a pre-operative FNA cytological description of TIR 3b, TIR 4, or TIR 5 according to the “Italian consensus for reporting thyroid fine-needle aspiration cytology” (ICCRTC) classification; “*” indicates valid percentages of patients with at least one cytologically suspicious thyroid nodule at pre-operative FNA, calculated by excluding missing data. For patients with available data, the relative percentages of thyroid nodules categorized as TIR 3b, TIR 4, or TIR 5 are indicated; ETE: extrathyroidal extension; RAI: radioactive iodine therapy; TKI: tyrosine kinase inhibitors; PTC: papillary thyroid carcinoma; FTC: follicular thyroid carcinoma; HCC: Hürthle cell carcinoma. “**” indicates the tall-cell, columnar, hobnail, and solid-aggressive subtypes of PTC.

**Table 2:** Response to DTC therapy according to the pre-operative HT status.

Response to DTC Therapy	6 Months			12 Months			24 Months			>24 Months *		
	HT Negative (*n* = 182)	HT Positive (*n* = 77)	*p*	HT Negative (*n* = 161)	HT Positive (*n* = 66)	*p*	HT Negative (*n* = 142)	HT Positive (*n* = 50)	*p*	HT Negative (*n* = 125 †)	HT Positive (*n* = 40)	*p*
Excellent, *n*	107 (58.8%)	35 (45.5%)	**<0.001**	127 (78.9%)	55 (83.3%)	**0.029**	117 (82.4%)	44 (88.0%)	0.145	101 (80.8%)	38 (95.0%)	0.509
Biochemically incomplete, *n*	29 (15.9%)	7 (9.1%)		15 (9.3%)	1 (1.5%)		9 (6.4%)	0 (0%)		9 (7.2%)	0 (0%)	
Indeterminate, *n*	34 (18.7%)	35 (45.4%)		10 (6.2%)	9 (13.6%)		8 (5.6%)	5 (10.0%)		8 (6.4%)	1 (2.5%)	
Structurally incomplete, *n*	12 (6.6%)	0 (0%)		9 (5.6%)	1 (1.5%)		8 (5.6%)	1 (2.0%)		6 (4.8%)	1 (2.5%)	
Persistence of indeterminate response, *n*				5/34 (14.7%)	7/35 (20.0%)	0.752	4/10 (40.0%)	3/9 (33.3%)	0.999			
New onset indeterminate response, *n*				5/10 (50.0%)	2/9 (22.2%)	0.349	4/8 (50.0%)	2/5 (40.0%)	0.999			

“*” refers to the last post-operative visit for the subset of patients with more than 24 months of follow-up (median follow-up, 5 years); “†” indicates that one patient died during follow-up. Bold values denote statistical significance at the *p* < 0.05 level.

**Table 3:** Logistic regression analysis.

	Standardized β	Odds Ratio	95% CI	*p*
Male sex	0.466	13.355	1.551–114.994	0.018
Age at diagnosis	−2.092	1.030	0.967–1.113	0.306
Tumor size	2.592	0.976	0.906–1.052	0.529
HT-positive status	1.086	2.962	0.512–17.132	0.225
Adjuvant RAI	−1.041	0.353	0.047–2.644	0.311

The odds ratio was adjusted by forcing the tumor histological characteristics in the logistic regression model. Multicollinearity of predictors was tested via the evaluation of variance inflation factors (VIFs). All VIF measures were <2.5. The bold value denotes statistical significance at the *p* < 0.05 level.

## Data Availability

The data supporting the reported results are available from the corresponding author upon request.
